# Detection of enterovirus RNA in pancreas and lymphoid tissues of organ donors with type 1 diabetes

**DOI:** 10.1007/s00125-025-06359-w

**Published:** 2025-03-17

**Authors:** Jutta E. Laiho, Sami Oikarinen, Sofia Morfopoulou, Maarit Oikarinen, Ashlie Renner, Daniel Depledge, Matthew C. Ross, Ivan C. Gerling, Judith Breuer, Joseph F. Petrosino, Vincent Plagnol, Alberto Pugliese, Antonio Toniolo, Richard E. Lloyd, Heikki Hyöty, for the nPOD-Virus Group

**Affiliations:** 1 Department of Virology, Faculty of Medicine and Health Technology, Tampere University, Tampere, Finland; 2 Department of Infection, Immunity and Inflammation, UCL Great Ormond Street Institute of Child Health, University College London, London, UK; 3 Department of Molecular Virology and Microbiology, Baylor College of Medicine, Houston, TX USA; 4 Alkek Center for Metagenomics and Microbiome Research, Baylor College of Medicine, Houston, TX USA; 5 Division of Endocrinology, Diabetes, and Metabolism, Department of Medicine, University of Tennessee Health Science Center, Memphis, TN USA; 6 Genomics PLC, Oxford, Oxfordshire UK; 7 Department of Diabetes Immunology, Arthur Riggs Diabetes & Metabolism Research Institute, Beckmann Research Institute, City of Hope, Duarte, CA USA; 8 Global Virus Network, University of Insubria, Varese, Italy; 9 Fimlab Laboratories, Tampere, Finland; 10 Department of Pediatrics, Tampere University Hospital, Tampere, Finland

**Keywords:** Enterovirus, Islet autoimmunity, Lymph nodes, Organ donors, Pancreas, Persistent infection, Spleen, Type 1 diabetes

## Abstract

**Aims/hypothesis:**

The nPOD-Virus group collaboratively applied innovative technologies to detect and sequence viral RNA in pancreas and other tissues from organ donors with type 1 diabetes. These analyses involved the largest number of pancreas samples collected to date. The aim of the current work was to examine the presence of enterovirus RNA in pancreas and lymphoid tissues of organ donors with and without type 1 diabetes.

**Methods:**

We analysed pancreas, spleen, pancreatic lymph nodes and duodenum samples from the following groups: (1) donors with type 1 diabetes (*n*=71) with (*n*=35) or without (*n*=36) insulin-containing islets; (2) donors with single or double islet autoantibody positivity without diabetes (*n*=22); and (3) autoantibody-negative donors without diabetes (control donors) (*n*=74). Five research laboratories participated in this collaborative effort using approaches for unbiased discovery of RNA viruses (two RNA-Seq platforms), targeted detection of *Enterovirus A–D* species using RT-PCR, and tests for virus growth in cell culture.

**Results:**

Direct RNA-Seq did not detect virus signal in pancreas samples, whereas RT-PCR detected enterovirus RNA confirmed by sequencing in low amounts in pancreas samples in three of the five donor groups: donors with type 1 diabetes with insulin-containing islets, 16% (5/32) being positive; donors with single islet autoantibody positivity, 53% (8/15) being positive; and non-diabetic donors, 8% (4/49) being positive. Detection of enterovirus RNA was significantly more frequent in single islet autoantibody-positive donors compared with donors with type 1 diabetes with insulin-deficient islets (*p*<0.001) and control (non-diabetic) donors (*p*=0.004). In some donors, pancreatic lymph nodes were also positive. RT-PCR detected enterovirus RNA also in the spleen of a small number of donors and virus enrichment in susceptible cell lines before RT-PCR resulted in much higher rate in spleen positivity, particularly in donors with type 1 diabetes. Interestingly, the enterovirus strains detected did not cause a typical lytic infection, possibly reflecting their persistence-prone nature.

**Conclusions/interpretation:**

This was the largest coordinated effort to examine the presence of enterovirus RNA in the pancreas of organ donors with type 1 diabetes, using a multitude of assays. These findings are consistent with the notion that donors with type 1 diabetes and donors with islet autoantibodies may carry a low-grade enterovirus infection in the pancreas and lymphoid tissues.

**Graphical Abstract:**

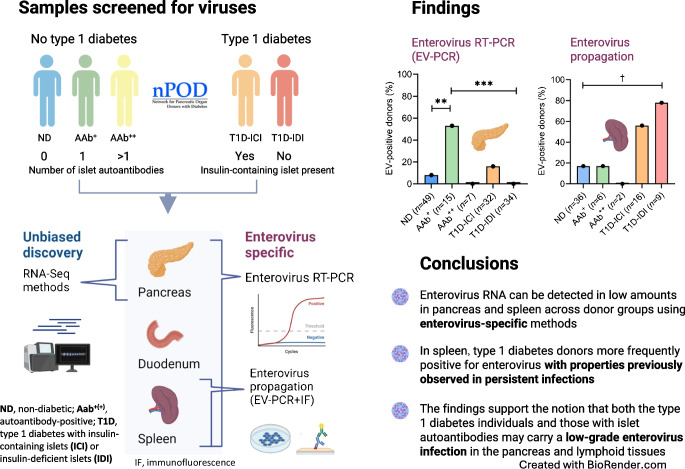

**Supplementary Information:**

The online version of this article (10.1007/s00125-025-06359-w) contains peer-reviewed but unedited supplementary material.


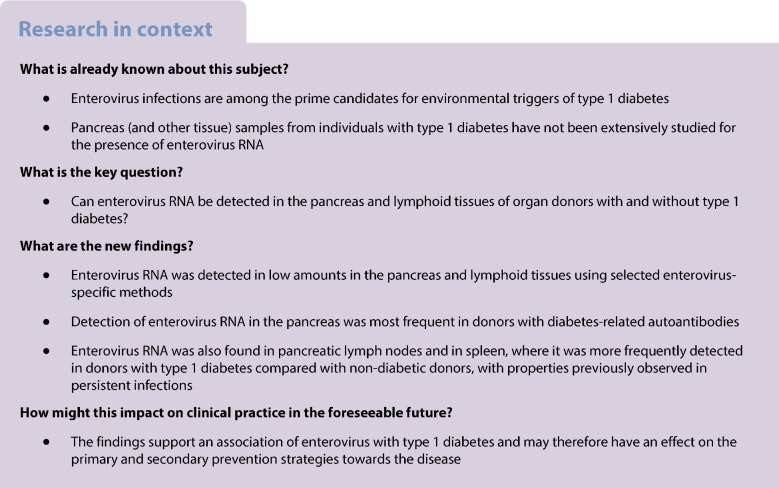


## Introduction

The presence of enterovirus viral capsid protein 1 (VP1) in pancreatic islets [1–3], mostly in beta cells, has been associated with type 1 diabetes; some studies have also found enterovirus RNA in the islets [3–6]. Studies of infants who died from acute enterovirus infections demonstrated virus in pancreatic islets, with inflammation and cell damage [7, 8]. The DiViD study examined pancreatic tissue obtained at biopsy from six newly diagnosed adults and found evidence of enterovirus infection in the islets of all six [9]. Thus, enteroviruses have tropism for pancreatic islet cells, including beta cells. This has also been observed in cell models wherein enteroviruses readily infect human beta cells and impair insulin secretion [10]. Such tropism to beta cells could be explained by the abundant expression of coxsackie and adenovirus receptor (CAR) [11], which is used by the coxsackie B group of enteroviruses linked to type 1 diabetes [12]. The reduced ability of beta cells to generate innate immune responses may contribute to their susceptibility to viral infection [13] and perhaps limits their ability to fully clear the infection.

Previous studies suggested that enteroviruses can undergo terminal deletion in myocardium and pancreas, becoming replication-defective and favouring persistence as a low-grade chronic infection [14, 15] that may be less susceptible to immune clearance. Prolonged, persisting infection by replication-defective viruses could explain the high detection rate of enterovirus protein or RNA in the pancreatic islets of individuals with type 1 diabetes (80–100% of individuals) and the challenge of isolating replication-competent virus from their pancreas [9]. In addition, very few beta cells are positive for enterovirus protein and the levels of viral RNA are extremely low in the pancreatic islets. This does not fit with a typical acute phase of infection where usually several cells are infected, producing large amounts of virus. In addition to the pancreatic islets of individuals with type 1 diabetes, some studies have detected enteroviruses in the small intestinal mucosa, a well-known enterovirus replication site [16–18].

The Network for Pancreatic Organ Donors with Diabetes (nPOD) has collected pancreas and other tissue specimens from organ donors with type 1 diabetes through a wide spectrum of age and disease duration. The nPOD-Virus Group has been established as an international collaboration to investigate viral infections in pancreas and other tissues. Here, we report the group efforts focused on detecting and sequencing viral RNA in pancreas, spleen, pancreatic lymph nodes (PLNs) and duodenum. Independent laboratories in Finland, Italy, the USA and the UK developed and applied, for the first time, RNA-Seq methods for the unbiased discovery of any RNA virus potentially present in the pancreas of individuals with type 1 diabetes. Based on the pre-existing association data with enteroviruses, the group also deployed highly sensitive, enterovirus-specific detection methods to directly address the presence of enterovirus RNA.

## Methods

### Organ donors and tissues

We examined cadaveric organ donor tissue samples collected by nPOD. As part of the coordinated efforts of the nPOD-Virus Group, we investigated tissues from 167 organ donors: 71 with type 1 diabetes, of whom 35 had residual insulin-containing islets (ICIs), comprising the T1D-ICI group, and 36 only had insulin-deficient islets (IDIs), comprising the T1D-IDI group; 22 were islet autoantibody (AAb) positive without diabetes considered at increased risk for type 1 diabetes, of whom 15 expressed a single AAb (AAb^+^ group) and seven had two or more AAbs (AAb^++^ group); and 74 were negative for AAbs, included as a control group (non-diabetic [ND]). Demographic information for each group is summarised in Table 1. The sex of the organ donors was provided in the medical charts. Detailed donor information is provided in ESM Table 1. The standardised collection protocol for the tissues analysed is described in Campbell-Thompson et al [19].

**Table 1:** Donor demographics for each of the five donor groups

Donor group	ND	AAb^+^	AAb^++^	T1D-ICI	T1D-IDI
Total, *N*	74	15	7	35	36
Age, years	19.6 (9.9–38.1)	25.2 (18.8–41.4)	23.0 (22.0–40.3)	20.0 (13.0–24.9)	28.9 (19.7–36.0)
Age range, years	0.3–75.0	2.2–64.8	17.7–69.2	5.0–45.0	4.4–78.0
Sex, *n* male/*n* female (% male sex)	45/29 (60.8)	10/5 (66.7)	5/2 (71.4)	17/18 (48.6)	19/17 (52.8)
BMI, kg/m^2^	23.9 (18.7–28.5)	22.2 (18.8–27.0)	26.3 (21.3–29.7)	24.0 (20.5–26.3)	24.5 (22.9–26.7)
Duration of disease, years	NA	NA	NA	5.0 (2.0–9.0)	14.0 (8.0–28.0)
Duration range, years	NA	NA	NA	0–32.5	1.5–74
C-peptide, ng/ml	1.29 (0.62–2.39)^a^	1.26 (0.58–3.31)	1.80 (0.61–5.50)		
Detectable C-peptide, *n* (%)				17 (48.6)	2 (5.5)

Data are presented as median (IQR) unless stated otherwise

^a^Information available from 68/74 donors

Frozen pancreas samples were analysed for the majority of organ donors. For selected donors, other organs were recovered (spleen, PLNs, live cryopreserved lymphoid cells and duodenal mucosa). Samples were stored at −70°C to −80°C. All samples were de-identified and obtained by nPOD through its partnership organ procurement organisations, after consent for organ donation and research was obtained from family members and the Health Center Review Board, University of Florida. Frozen samples were shipped by air to participating laboratories in dry ice or using small liquid-nitrogen containers. On arrival, samples were stored at −70°C to −80°C until used.

**Fig. 1:**
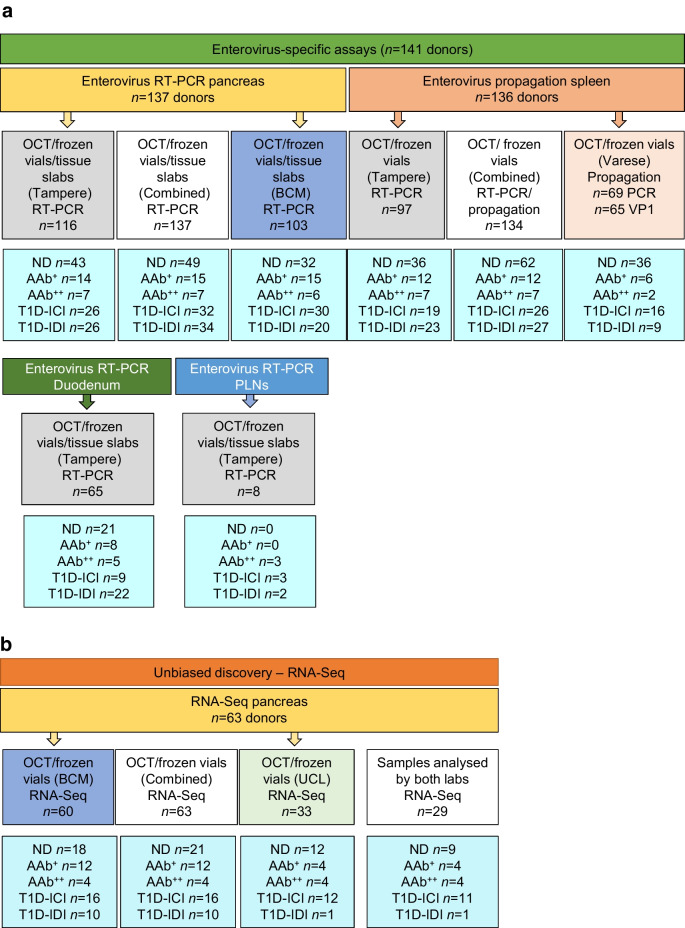
Study design and distribution of samples for analyses. Sample allocation depended on the assay performed; however, for each assay, samples were distributed in various batches. Samples were either snap-frozen tissue pieces, live lymphoid cells or tissue pieces frozen in optimal cutting temperature (OCT) compound. (**a**) The number of donors analysed using enterovirus-specific assays at BCM and Tampere laboratories (enterovirus-specific RT-PCR) and Varese laboratory (enterovirus propagation in cell cultures). The samples analysed in the laboratories combined together are indicated in white boxes. (**b**) Number of donors examined by unbiased discovery (RNA-Seq) at BCM and UCL laboratories. The samples analysed in the laboratories combined together are indicated in white boxes

Five different laboratories performed independent assays using diverse methodologies to detect traces of enteroviruses or other microbes in pancreas and other tissues. A goal of the nPOD-Virus group was to approach the question about viral aetiology of type 1 diabetes and explore what type of viruses may be present and, if so, their potential association with disease. To this end, we implemented two unbiased discovery approaches for microbes, based on two different RNA-Seq methods. In addition, based on pre-existing evidence of an association of type 1 diabetes with enterovirus infections, we employed enterovirus-specific RT-PCR assays, and enterovirus propagation in cell cultures, followed by RT-PCR as well as enterovirus capsid protein staining. Samples from the donors were distributed to the five participating laboratories according to the protocol shown in Fig. 1.

### Unbiased discovery of microbes

RNA-Seq studies were performed on pancreas samples in two laboratories, one at University College London (UCL), London, UK, and the other at Baylor College of Medicine (BCM), Houston, USA. Based on sample availability for coordinated studies, RNA-Seq analyses were performed on pancreas samples from 63 nPOD donors: 16 T1D-ICI; ten T1D-IDI; four AAb^++^, 12 AAb^+^; and 21 ND. Of these 63 donors, 29 were analysed in both laboratories (11 T1D-ICI, one T1D-IDI, four AAb^++^, four AAb^+^, nine ND).

#### RNA-Seq analyses at UCL

Over 4 years, UCL sequenced frozen pancreas samples from 33 nPOD cases (12 T1D-ICI, one T1D-IDI, four AAb^+^, four AAb^++^ and 12 ND). We developed the methodology in four steps (described below) and used several extraction and library preparation approaches to maximise sensitivity. Initial negative results motivated the development of a specific sequence capture method [20] to enrich for enteroviral sequences and addition of the analysis of laser-captured islet RNA to further increase sensitivity [21]. The RNA obtained was then subjected to Illumina high-throughput RNA sequencing.

In step I (first pilot stage), we examined pancreas from three T1D-ICI, one T1D-IDI and two ND donors, based on availability of optimal samples for RNA-Seq. Disease duration ranged from 4 years to 28 years.

In step II, we investigated tissues from donors with shorter disease duration to minimise the time between sample collection and type 1 diabetes onset (four T1D-ICI donors [disease duration range 1–5 years], four AAb^++^ and three AAb^+^ donors).

In step III we examined pancreas from four T1D-ICI donors with enterovirus VP1 immuno-positivity by immunohistochemistry and HLA class I hyperexpression, along with five ND donors (for details, see immunohistochemistry results in the accompanying publication by Rodriguez-Calvo et al [22]). From one donor with type 1 diabetes, two samples were analysed. Finally, in step IV, we examined laser microdissected islets from six T1D-ICI, four AAb-positive (three AAb^++^, one AAb^+^) and six ND donors. From one donor with type 1 diabetes, two samples were analysed.

In steps I and II, total RNA was isolated using Illumina GAIIx (step I) or the Illumina HiSeq2500 (step II), followed by a poly(A) selection step for mRNA. In steps III and IV, we used the Agilent SureSelect system to enrich the potential enteroviral sequences in pancreatic samples. RNA extraction was performed as described [23]. For double-stranded (ds) cDNA generation, we used a protocol optimised for RNA viruses [20, 24]. The ds-cDNA was sheared, and libraries prepared as per the SureSelect protocol v1.4. Enrichment for enteroviral sequences was performed using a set of 120-mer biotinylated RNA oligonucleotides prior to indexing and sequencing on different Illumina platforms (MiSeq, HiSeq, NextSeq). The bait set (RNA oligonucleotides) was designed using an in-house script written for a European Union-funded project aimed at using SureSelect in a pathogen diagnostic setting (PathSeek). The bait set hybridised against all members of the enterovirus A (*n*=363 probes), B (*n*=176) and C species (*n*=303), based on sequences available in GenBank at the time of design (15 May 2013). Up to eight mismatches in a 120-mer oligo was accepted to still enable capture of the targeted sequence, ensuring enterovirus detection provided these shared a reasonable degree of similarity.

#### Positive control experiment for sequence capture

As positive control, ULC sequenced pancreatic tissue samples that were spiked in at different dilutions (1×10^−4^ to 1×10^−8^ range) of coxsackie B virus 1 (CVB1) and a negative control, to assess the sensitivity of the sequence capture method prior to its use (ESM Methods).

#### Metagenomic whole-genome shotgun sequencing at BCM

BCM performed metagenomic whole-genome shotgun (WGS) sequencing from 60 nPOD frozen pancreas samples (16 T1D-ICI, ten T1D-IDI, 12 AAb^+^, four AAb^++^ and 18 ND). Total pancreatic nucleic acids were extracted using the MagMax Viral RNA Isolation Kit (catalogue no. AM1939, Thermo Fisher, Waltham, MA, USA), without DNAse to prevent DNA removal. Extracted viral RNA was reverse transcribed using SuperScript II RT (catalogue no. 18064014, Thermo Fisher) and random hexamers. After short molecule and random hexamer removal with ChargeSwitch (catalogue no. CS12000, Thermo Fisher), molecules were amplified and tagged with a 12-base-pair barcode tag containing a V8A2 semi-random primer (BC12-V8A2 construct using AccuPrime Taq polymerase and cleaned with a ChargeSwitch kit). Tags were attached via a barcoded, semi-random primer construct resulting in dual barcoded (same barcode on both sides) amplified fragments. The indexes used were 12 bp Golay Barcodes. Separate negative controls were introduced during extraction, amplification and library preparation steps. We performed a single WGS library preparation per sequencing lane (without shearing) of pooled, pre-barcoded samples to minimise carry-over, as each lane only had a single index. Since all samples carried secondary internal barcodes, they were not subject to carry-over or cross-bleed, as sometimes is observed from run to run with library indexes using the Illumina platform. The size of the library was verified via Illumina Bioanalyzer to ensure appropriate range for the platform (~200–1000 bp). The library was then loaded in an Illumina HiSeq2000 (Illumina, Carlsbad, CA, USA) and sequenced using the 2×100 bp chemistry at the Human Genome Sequencing Center, BCM. Reads were demultiplexed into a sample bin using the barcode prefixing read-1 and read-2, allowing zero mismatches. Demultiplexed reads were further processed by trimming off barcodes, semi-random primer sequences and Illumina adapters. This process used a custom demultiplexer and the BBDuk algorithm included in BBMap53.

#### Bioinformatics analysis and community profiling at UCL and BCM

A dual analytic approach was implemented. Data were first analysed in an unbiased manner, assuming no prior knowledge of potential pathogens and characterising the full species profile for each sample. In addition, data were specifically searched for enteroviral sequences. PCR duplicates were removed with an in-house script that collapses read pairs by sequence identity using 90% of the sequence as signature. We removed low quality and low complexity sequences with PrinSeq [25] and human sequences with Novoalign (version V2.07.13 - human reference genome GRCh37) followed by BLASTn [26]. High-quality contigs of at least 200 bp length were de novo assembled with Velvet [27]. Contigs and the unassembled reads were annotated with BLASTx (default parameters) and a custom protein database consisting of viral, human microbiome bacterial, human and mouse RefSeq proteins (October 2013 version). Coxsackievirus proteins that were not present in the RefSeq collection were added to the database. To search specifically for enterovirus sequences, we aligned (Novoalign V2.07.13) quality-controlled reads simultaneously to the genomes of enteroviruses from species A, B and D (NC_001612, NC_001472, NC_001430). This search was repeated using all enterovirus full genomes from GenBank (221 genomes, January 2020) and Bowtie2 [28]. We employed metaMix 0.1 [29], which is used in clinical diagnostics for pathogen detection in brain biopsies from individuals with encephalitis of unknown cause [30–35], to characterise the species that were present in each sample. The read support parameter cutoff for a species to be retained in the profile was ten reads.

### Targeted enterovirus detection by RT-PCR

The presence of enterovirus RNA was assessed in tissues from 141 nPOD organ donors using a sensitive RT-PCR assay. Frozen pancreas samples from 137 nPOD organ donors (32 T1D-ICI, 34 T1D-IDI, seven AAb^++^, 15 AAb^+^, 49 ND) were analysed in two laboratories, one at Tampere University, Finland and one at the Department of Molecular Virology and Microbiology, BCM, Houston, TX, USA. Based on sample availability, the Tampere laboratory also examined frozen spleen samples from 97 organ donors (19 T1D-ICI, 23 T1D-IDI, seven AAb^++^, 12 AAb^+^ and 36 ND), PLN samples from eight organ donors (three T1D-ICI, two T1D-IDI and three AAb^++^) and duodenum samples from 65 organ donors (nine T1D-ICI, 22 T1D-IDI, five AAb^++^, eight AAb^+^ and 21 ND).

In Tampere, RNA was extracted from frozen tissue using the Viral RNA Kit (Qiagen, Hilden, Germany) and samples were analysed with a quantitative real-time RT-PCR method [36]. In the BCM laboratory, pancreatic RNA was extracted with the MagMax Viral RNA Isolation Kit (Invitrogen; Thermo Fisher). RNA was converted to cDNA with Superscript III RT (Invitrogen) according to the manufacturer’s directions, with random primers. PCR was carried out with SYBR-Green PCR master mix (Invitrogen) using the same primers as in Tampere [36]. PCR included a denaturation step (95°C for 10 min) followed by 50 cycles of 95°C for 30 s and 60°C for 60 s. In both laboratories, positive RT-PCR signals were confirmed by sequencing the PCR amplicon and samples were considered positive only if an enterovirus sequence was obtained.

The degree of RNA degradation was analysed in selected pancreas, spleen and duodenum samples, using Agilent Fragment Analyzer.

### Enterovirus propagation in cell culture prior to RNA detection by RT-PCR

Enterovirus propagation in cell culture was carried out for spleen samples at the University of Insubria, Varese, Italy, to amplify the virus prior to RT-PCR assays and immunostaining. For this approach we selected spleen samples since they do not contain enzymes that can affect cultured cells. Donors were selected according to the availability of live spleen cell suspensions for both control donors and donors with type 1 diabetes. Occasionally, snap-frozen spleen tissue was also tested in the form of spleen homogenates. We could examine samples from 69 donors (16 T1D-ICI, nine T1D-IDI, two AAb^++^, six AAb^+^, 36 ND). A published procedure for detecting persistent enterovirus infections [9, 37] was followed, with minor modifications. Briefly, to enrich for virus nPOD spleen samples (live cells or tissue homogenates) were co-cultured in T-25 flasks with five different human cell lines (AV3, RD, 1.1B4, VC3, HEK-293; European Collection of Authenticated Cell Cultures, Porton Down, UK) that express a wide range of enterovirus receptors. Human cell lines were grown in DME/F12 medium supplemented with penicillin/gentamicin and with 10% (vol./vol.) heat inactivated FBS (Gibco; Thermo Fisher, Rodano, Italy). Cultured cells were checked monthly for mycoplasma contamination (MycoAlert Plus Mycoplasma kit; Euroclone-Lonza, Pero, Italy). For immunofluorescent detection of enterovirus VP1 antigen, cell cultures were prepared in Millicell EZ 4-well glass slides (Merck, Vimodrone, Italy), as described below. At the third passage, the supernatant fraction of cell cultures was used for RNA extraction and RT-PCR. Extracted RNA was reverse transcribed and enterovirus-specific endpoint PCR assays were performed using five different primer sets. Capillary electrophoresis (Agilent 2100 Bioanalyzer, Milano, Italy) was used to detect the precise size of amplicons whose sequences were obtained by the Sanger method. For indirect immunofluorescence, cell monolayers were fixed in PBS containing 4% paraformaldehyde. Enterovirus-infected cells were spotted by staining with two different mouse monoclonal antibodies against the VP1 enteroviral capsid antigen (9D5 from Merck; 6-E9/2 from Creative Diagnostics). The two antibodies bind to distinct stretches of the VP1 protein. Both recognise acute and persistent enterovirus infection in cultured cells, are devoid of neutralising activity, and are specific for a vast spectrum of enterovirus types. The 9D5 antibody binds to the consensus motif SIGNAYSMFYDG [38] while 6-E9/2 recognises the same epitope of the 5D8/1 antibody [39]. Alexa Fluor 488-goat anti-mouse IgG was used as secondary antibody.

### Statistical analysis

Statistical analyses were performed using SPSS 25.0 for Windows. Frequency comparisons was performed with the Pearson’s χ^2^ and Fisher’s exact tests. When comparing donor groups, the significant *p* values were corrected for multiple comparisons by multiplying the raw *p* value by the number of comparisons made (Bonferroni’s correction).

## Results

### Metagenomic sequencing did not detect viruses or other microbes in the pancreas

In the CVB1-spiked quality control samples, the RNA was of high integrity and stability. CVB1 reads were detected with all sequencing approaches used, even at the lowest dilution (1×10^−8^), confirming high sensitivity of the assays. There was a linear relationship between the spiked-in virus dilution level and the CVB1 reads count (ESM Fig. 1; ESM Table 2). In the negative control, we detected one pair of CVB1 reads; this does not meet the species detection criteria and was considered to be possible low-level cross-contamination from the highest viral load samples.

Most pancreas samples used at UCL for metagenomic steps I–IV sequence analyses had moderately to highly degraded RNA. The search for enteroviral reads did not yield positive hits in any of the analysis steps. In steps I and II, the deduplicated reads were mostly human sequences. Taxonomic classification of the ‘microbial’ reads with metaMix resulted in a similar profile for all samples, showing no difference between case and control donors. Most reads were assigned to enterobacteria phage phiX174, the positive control for Illumina sequencing. The rest of the reads were divided between various environmental bacteria and the ‘unknown’ bin (ESM Table 3). In step III, which included a specifically designed enterovirus enrichment method, the reads were highly clonal and only a small fraction of the data was non-human. Community profiling identified human and enterobacterial phage phiX sequences, and most of the small number of ‘microbial’ reads were unassigned (for a representative summary, see ESM Table 4). In two out of ten samples, there were a few reads from human herpesvirus 3. We consider that this was likely to be a contaminant, as this virus is frequently sequenced on the Illumina machines at UCL.

In the RNA extracted from laser microdissected islets (step IV analyses with the targeted enterovirus baits), the number of ‘microbial’ reads was low, despite the high sequencing depth. The metaMix profile consisted of two or three species per sample and there was low-level carry-over contamination with viruses routinely sequenced at UCL. To rule out the remote possibility that unassigned reads originate from a species not present in our database, a BLASTn search was performed against the nucleotide-NR database. The reads remained unclassified and only a few thousand reads matched human, bacterial or uncultured eukaryote sequences.

At BCM, 68 pancreatic tissue samples were sequenced, including pooled laser-captured islets from three samples, to amplify islet-specific signal in selected donors but no virus sequences were detected.

### Detection of enterovirus RNA using sensitive RT-PCR assays

**Fig. 2:**
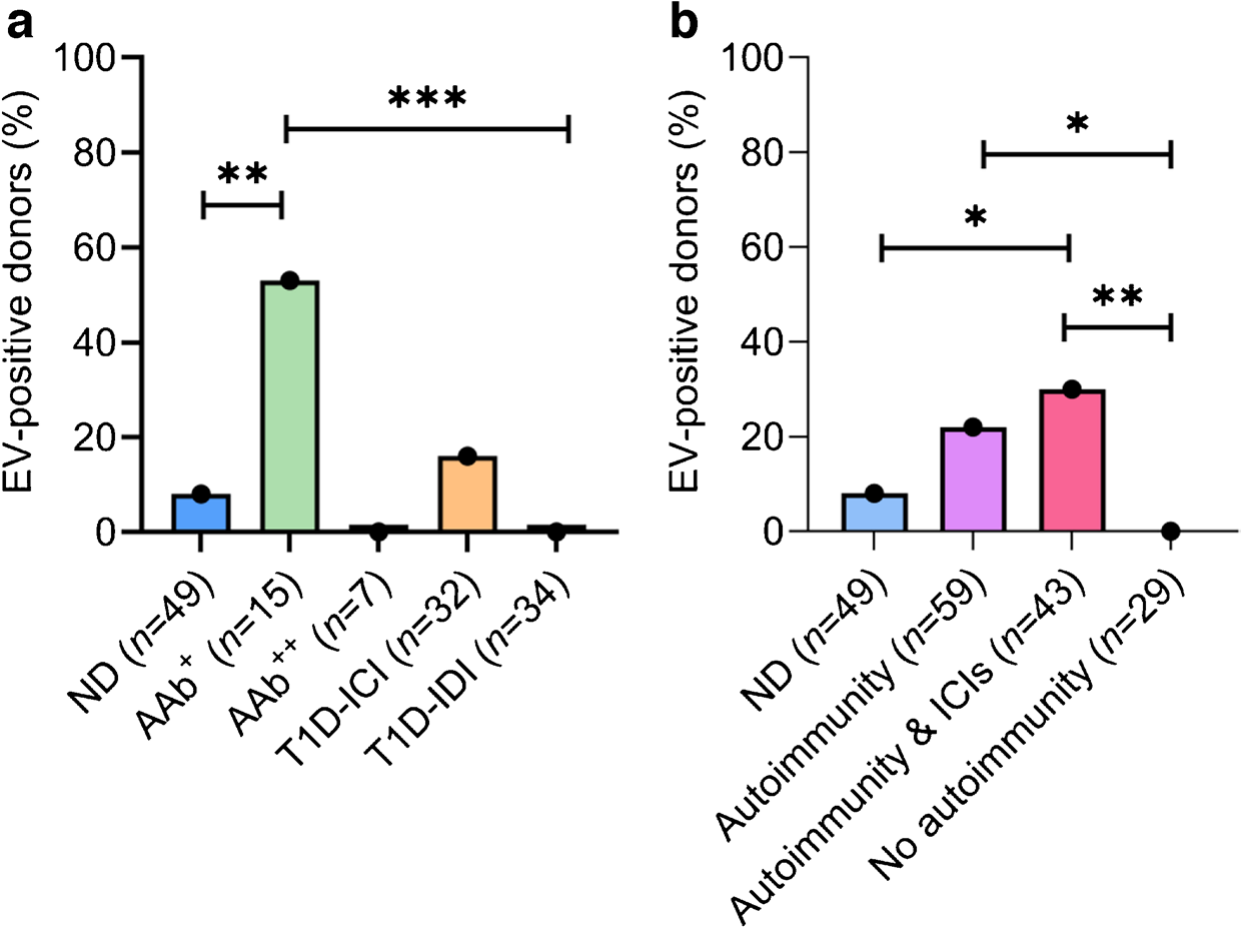
Enterovirus RNA was detected in pancreas with RT-PCR. (**a**) Enterovirus RNA was detected in the pancreas across different donor groups. The detection rate was higher in AAb^+^ donors compared with ND donors and T1D-IDI donors. **p<0.01, ***p<0.001 (corrected for multiple [*n*=10] comparisons). Other corrected group comparisons were non-significant. (**b**) Donors with active islet autoimmunity (‘Autoimmunity’), as marked by AAb positivity, regardless of type 1 diabetes status, had a higher prevalence of enterovirus RNA positivity, especially those with residual beta cells (‘Autoimmunity & ICIs’), compared with donors with type 1 diabetes without active autoimmunity (‘No autoimmunity’) and ND control donors. *p<0.05, **p<0.01 (corrected for multiple [*n*=6] comparisons). EV, enterovirus

Analyses of pancreas samples from 137 donors by sensitive RT-PCR methods detected enteroviruses in three of the five donor groups, and most frequently in the AAb^+^ donors. A sample was considered positive if at least one of the two laboratories detected enterovirus RNA and the sequence of the PCR product matched with enterovirus. Taken together, enterovirus was detected in the pancreas in 16% (5/32) of T1D-ICI donors, 0% (0/34) of T1D-IDI donors, 0% (0/7) of AAb^++^ donors, 53% (8/15) of AAb^+^ donors and 8% (4/49) of ND donors (Fig. 2a). AAb^+^ donors had a significantly higher frequency of positivity than T1D-IDI (*p*<0.001, corrected for multiple comparisons) and ND donors (corrected *p* value=0.004) (see also ESM Table 5). Sequencing of the RT-PCR products identified variations in the amplified genome region, implying the presence of different enterovirus genotypes across donors. Based on the obtained sequences, over 90% of them belonged to *Enterovirus B* species. However, since the amplified region locates within the conserved part of the viral genome, the identification of the exact genotype of these viruses was not possible. The sequences obtained from all the samples are listed in Table 2.

**Table 2:** Examples of enterovirus nucleotide sequences detected in the samples by enterovirus-specific RT-PCR and their alignment to different enterovirus species

Donor ID	Donor type	Tissue	Sequence	Species
6024	ND	Pancreas	TCCTAACTGCGGAGCACACACCCTCAAACCAGAGGGCAGTGTGTCGTAACGGGCAACTCTGCAGCGGAACCGAT	EV B
6029	ND	Pancreas	TCCTAACTGCGGAGCAGATACCCACACACCAGTGGGCAGTCTGTCGTAATGNNCAACTCTGCAGCGGAACCAAC TCCTAACTGCGGAGCAGATACCCACACACCAAGTGGGCAGTCTAGTCGTAATAGGGCAACTCTGCAGCGGAACCGAC	EV B
6097	ND	Spleen	TTCCAACCTCGGGGCAGGTGTCACAAAACCAGTGTATGGCCTGTCGTAACGCGCAAGTCCCGTGGCGGAACCGAC	EV C
6112	ND	Spleen	TCTTAACCATGGAGCAAGTGCTCACAAGCCAGTGAGTTGCTTGTCGTAMMGCGCAAGTGCCGTGGCGGAACCGA	EV D
6168	ND	Pancreas	TCCTAACTGCGGAGCACATACCCTCAAGCCAGAGGGCAGTGTGTCGTAATGGGCAACTCTGCAGCGGAACCGAC	EV B
6182	ND	Pancreas	TCCTAACTGCGGAGCACATACCCTCAAACCAGGGGCGTGTGTCGTACGGGCACTCTGCAGCGGAACCGAC	EV B
6044	AAb^+^	Pancreas	TCCTAACTGCGGAGCAGGTACTCACGAACCAGTGGGCAGCCTGTCGTAACGGGCAACTCTGCAGCGGAACCGAC TCCTAACTGCGGAGCAGATACCCACATACCAGTGGGCAGTCTGTCGTAATGGGCAACTCTGCAGCGGAACCGAC	EV B
6101	AAb^+^	Pancreas	TCCTAACTGCGGAGCAGGCACTCACNATCCAGTGGGCAGCCTGTCGTAACGGGCAACTCTGCAGCGGAACCGAC	EV B
6123	AAb^+^	Pancreas	TCCTAACTGCGGAGCAGGTACCCACGAACCAGTGGGCAGTCTGTCGTAACGGGCAACTCTGCAGCGGAACCGAC TCCTAACTGCGGAGCACACACCCTCAAACTAGAGGGCAGTGTGTCGTAACGGGCAACTCTGCAGCGGAACCGAC TCCTAACTGCGGAGCACACACCTNAAACCAGAGGGCAGTGTGTCGTAACGGGCAATTCTGCAGCGGAACCGAT TCCTAACTGCGGAGCACACATCCTCAAACCAGAGGGCAGTGTGTCGTAACGGGCAACTCTNNAGCGGAACCGAN	EV B
6147	AAb^+^	Pancreas	TCCTAACTGCGGAGCAGGTACCCACGAACCAGTGGGCAGCCTGTCGTAACGGGCAACTCTGCAACGGAACCGAC	EV B
6154	AAb^+^	Pancreas	TTCTAACTGCGGAGCAGGTACCCACGAACCAGTGGGCAGCCTGTCGTAACGGGCACTCTGCAGCGGAACGA	EV B
6156	AAb^+^	Pancreas	TCTTAACTGCGGAGCAGGTACCTACGAACCAGTGGGCAGCCTGTCGTAACGGGCAACTCTGCAGCGGAACCGAC TCCTAACTGCGGAGCAGGTGCTCACAAACCAGTGAGTAGCCTGTCGTAATGGCCAACTCTGCAGCGGAACCGAC	EV B EV B
6181	AAb^+^	Pancreas	TCCTAACTGCGGAGCACATACCCTCAAACCAGGGGCAGTGTGTCGTAACGGGCAACTCTGCAGCGGAACCGAC	EV A
6184	AAb^+^	Pancreas	TCTGAGGCTAATTAGCAATAGATCGAGGAGCAGTGAGACGGTTGTCGTAATGCGTAAGTC	EV C
6158		PLN	TCCTAACTGCGGAGCACATACCCTCAAGCCAGAGGGCAGTGTGTCGTAACGGGCAACTCTGCAGCGGAACCGAC	EV B/EV A
6046	T1D-ICI	Pancreas	TCCTAACTGCGGAGCAGGTACCCACGAACCAGTGGGCAGCCTGTCGTAACGAGCAACTCTGCAGCGGAACCGAC TCCCAACCGCGGAGCACACGTTCGCAGCCAGCGAGTGGTGTGTCGTCACGGGCAACTCTGCGGCGGAACCGAC	EV B EV B
		Spleen	TCCTAACTGCGGAGCAGATACCCACGCACCAGTGGCGGTCTGTCGTAACGGGCACTCTGCAGCGGAACCGAC	EV B
6052	T1D-ICI	Pancreas	TCCTAACCACGGAGCAATCGCACACGACCCAGTGAGTAGGTTGTCGTAATGCGTAAGTCTGTGGCGGAACCGAC	EV C
		Spleen	TCCTAACTGCGGAGCGCACACCTTCAATCCAGGAGGCGGTGCGTCGTAATGGGATAACTCTGCAGCGGAACCGAC	EV A
6070	T1D-ICI	Pancreas	TCCGAACCACGGAGCAATCGCCCACGACCCAGTGGTTGTGGTGTCGTAATGCGTAAGTCTGTGGCGGAACCGAC	EV C
6198	T1D-ICI	Pancreas	TCCTAACTGCGGAGCAGATACCCACACACCAGTGAGCAGTCTGTCGTAATGGCAACTCTGCAGCGGAACCAAC	EV B
6209	T1D-ICI	Pancreas	TCCTAACTGCGGAGCAGATACCCACACACCAGTGGGCAGTCTGTCGTAATGGGCAACTCTGCAGCGGAACCGAC	EV B
		Spleen	TCCTAACTGCGGAGCACATACTCACAAGCCAGTGAGTGGTGTGTCGTAATGGGCAACTCTGCAGCGGAACCGAC	EV A
		PLN	TCCTAACTGCGGAGCACATACTCACAAGCCAGTGAGTGGTGTGTCGTAATGGGCAACTCTGCAGCGGAACCGAC	EV A
6087	T1D-IDI	Spleen	TCCTAACTGCGGAGCAGGCAATCACAATCCAGTGGGTATRMATGTCRTACYWACACTCCGCAGCGGAACCGAC	EV B

The amplified region locates within the conserved region of the viral genome and does not allow identification of the exact type of detected enteroviruses

EV, enterovirus

We then investigated whether the presence of enterovirus RNA in pancreas is associated with active islet autoimmunity, as suggested by the presence of circulating islet AAbs in the donors at the time of death. As a result, 22% (13/59) of the donors with active islet autoimmunity (those without diabetes expressing one or more AAbs and those with type 1 diabetes still expressing AAbs) carried enterovirus RNA compared with 0% (0/29) of donors with type 1 diabetes lacking AAbs (corrected *p* value=0.04) and 8% (4/49) of AAb^−^ ND donors (*p* value not significant). In addition, when taking only those donors with active islet autoimmunity and residual beta cells (including T1D-ICI, AAb^+^ and AAb^++^ donors), 30% (13/43) were enterovirus positive (corrected *p* value=0.047 vs ND donors; corrected *p* value 0.006 vs no islet autoimmunity) (Fig. 2b and ESM Table 6).

Paired pancreas and PLN samples from seven donors were available for examination of enterovirus RNA using RT-PCR. One T1D-ICI donor tested positive in the pancreas and two donors (one T1D-ICI and one AAb^++^) in the PLNs (the T1D-ICI donor was also positive in the pancreas). Five donors tested negative in both tissues (two T1D-ICI, two AAb^++^ and one T1D-IDI).

**Fig. 3:**
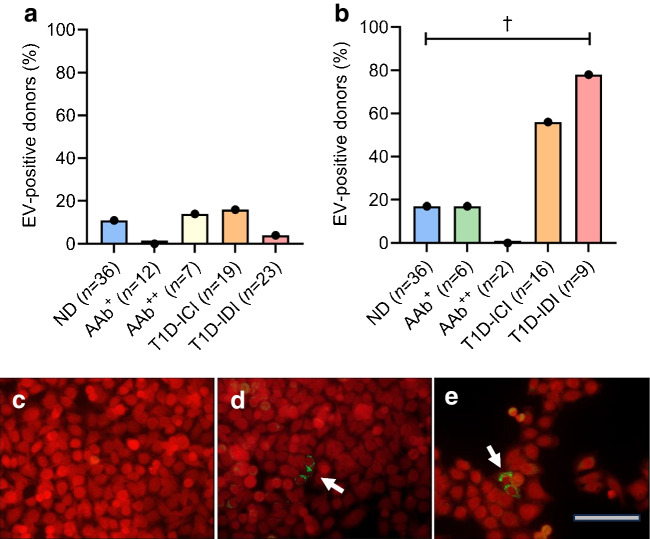
Enterovirus detection in spleen. (**a**) Spleen enterovirus RT-PCR. Enterovirus RNA was detected in 9/97 samples using RT-PCR followed by sequencing, without statistical significance between the donor groups. (**b**) Enterovirus propagation in cultured spleen cells. Combined results of the RT-PCR and immunostaining methods yielded 23/69 of the samples positive for enterovirus, with T1D-IDI donors being significantly more positive compared with ND donors. ^†^*p*=0.01 (Fisher exact test two-sided test, corrected for multiple [*n*=10] comparisons). (**c**–**e**) A representative example of immunostaining of cultured AV3 cells inoculated with spleen homogenates of one nPOD donor to visualise the production of enterovirus VP1 (mouse anti-enterovirus monoclonal antibody clone 6-E9/2, Creative Diagnostics). (**c**) Representative image of a cell culture inoculated by a spleen homogenate from an ND donor: production of viral protein is not observed. (**d**, **e**) Results obtained by spleen homogenates from two donors with type 1 diabetes showing the production of enterovirus protein in a few cultured cells (indicated by green colour and white arrows). Virus replication did not lead to a cytopathic effect typically observed in acute enterovirus infections. Magnification ×250. Scale bar, 50 µm. EV enterovirus

In spleen, enterovirus RNA was detected in 9% (9/97) of the donors (4/36 ND, 0/12 AAb^+^, 1/7 AAB^++^, 3/19 T1D-ICI, 1/23 T1D-IDI), with no statistically significant differences between groups (Fig. 3a and ESM Table 7). We did not detect enterovirus RNA in duodenal tissue of the 65 donors tested.

The quality of extracted RNA was studied in selected samples. The RNA quality number (RQN) values varied considerably between donors ranging from 1 (totally degraded RNA) to 10 (intact RNA). The median RQN value was 4.5 (range 1–7.8) in pancreas (*N*=12), 1.6 (1–6.2) in spleen (*N*=5) and 2.0 (1–7.3) in duodenum (*N*=10) samples. A low RQN score did not seem to affect the enterovirus PCR positivity since positive results were obtained also from samples with poor RNA quality (ESM Table 8). In all enterovirus-positive samples, virus loads were very low, often close to the detection limit of the assay.

### Detection of enterovirus in spleen using virus enrichment in human cell lines before RT-PCR

Sixty-nine spleen samples were analysed following virus amplification in cell culture prior to RT-PCR (*n*=69) and immunostaining steps (*n*=66). The criterium for positivity was an enterovirus-positive result for either one of the following methods: (1) for RT-PCR, C_t_ value below 32 plus amplicons of the expected size by capillary electrophoresis; and/or (2) cytoplasmic fluorescent staining in cultured cells. RT-PCR found positivity in 23/69 samples and immunostaining in 19/66 samples analysed. The agreement between methods was 97%, both being positive in 19 and negative in 46 of 66 samples analysed. Two samples were positive only by RT-PCR, in which case the result was deemed positive. Overall, enteroviruses were detected in 33% (23/69) of spleen samples. This was a significantly higher rate than that obtained by direct RT-PCR analysis of frozen spleen samples without virus propagation in cultured cells. In total: 56% (9/16) of T1D-ICI donors; 78% (7/9) of T1D-IDI donors; 0% (0/2) of AAb^++^ donors; 17% (1/6) of AAb^+^ donors; and 17% (6/36) of ND donors, were enterovirus positive using this protocol (Fig. 3b and ESM Table 9). T1D-IDI donors had significantly higher frequency of positivity compared with ND donors (*p*=0.01). T1D-ICI donors tended to be more frequently enterovirus positive than ND donors, although the difference did not reach statistical significance (*p*=0.069). When combining the direct RT-PCR from spleen with the detection of enterovirus replication in cell culture (replication detected by either RT-PCR or immunostaining, or both) (134 donors), T1D-ICI donors were more frequently positive for enteroviruses compared with ND donors, although the difference did not reach statistical significance (*p*=0.067). Enterovirus RNA was found in 39% (10/26) of T1D-ICI donors, 30% (8/27) of T1D-IDI donors, 14% (1/7) of AAb^++^ donors, 8% (1/12) of AAb^+^ donors and 13% (8/62) of ND donors. Importantly, enterovirus propagation from spleen cells yielded viruses that did not cause an evident cytopathic effect in cultured cells, unlike what is typically observed with replication-competent viruses that cause acute enterovirus infections. In cell cultures, only a few cells stained positive for enterovirus capsid protein VP1, indicating limited replication of the virus (Fig. 3c–e).

## Discussion

In the coordinated effort of the nPOD-Virus Group, we conducted the largest screening for RNA viruses and specifically enterovirus in the pancreas of organ donors with evidence of islet autoimmunity and/or type 1 diabetes. We employed multiple approaches to detect viral RNA. Studies aiming at detecting viral proteins are reported in companion publications [22, 40, 41]. For the first time in this setting we employed two metagenomic sequencing methods to broadly ascertain the possible presence of RNA viruses and, based on previous literature associations, we employed enterovirus-specific methods (RT-PCR followed by sequencing of PCR amplicons to detect enterovirus RNA, and in vitro virus propagation in cell culture followed by RT-PCR plus immunostaining to detect enterovirus replication).

We developed two non-biased RNA-Seq methods specifically to analyse organ donor pancreas; this approach has never been attempted before. Despite all the measures we took to maximise sensitivity and considering that our sequencing approaches had relatively high sensitivity in detecting enterovirus RNA in infected cell lines, both RNA-Seq methods, even when using sequence capture or RNA from laser-captured islets, failed to detect any viral RNA sequences. In a recent study, Manduchi et al faced similar technical limitations when applying an RNA-Seq method to detect enterovirus RNA in human pancreatic islets [42]. While we examined transplant-grade pancreases, we consider that several factors could have negatively impacted assay sensitivity to detect extremely low amounts of viral RNA: (1) signal loss due to RNA degradation in organ donor pancreas, an organ that is known to degrade rapidly; (2) the well-known rarity of infected cells, with associated sampling limitations, and thus likely suboptimal targeting of sampling; (3) the limited likelihood that we would detect evidence of viral infection at the time of death, since such an infection may have occurred years prior to disease development; and (4) the possible presence of replication-defective viruses producing too-small amounts of RNA copies [14, 43–45]. This experience provides a backbone for improvement for future studies likewise aiming at detecting viruses in pancreas.

In contrast, our highly sensitive RT-PCR assays detected enterovirus RNA in the pancreas across all donor groups. Even though the amount of virus was very low and close to the detection limit, two independent laboratories confirmed the presence of enterovirus RNA in the pancreas of 17 donors, overall. Enterovirus RNA was more prevalent in ND donors with a single AAb compared with control donors, possibly representing people at earlier stages of the disease progression, when enteroviruses are suggested to act as triggers and may be more likely to be present. Moreover, donors with signs of active autoimmunity, including being positive for islet AAb with or without type 1 diabetes, had higher prevalence of enterovirus RNA positivity than donors without active autoimmunity. Thus, it is possible that enterovirus infection in the pancreas is associated with ongoing autoimmune responses against beta cell antigens. The fact that the RNA virus load was extremely low fits with a low-grade, possibly persistent, infection rather that an acute infection. Beta cells may be particularly permissive for such prolonged/persistent infection due their relatively weak antiviral innate immune response [13] and high expression of CAR [11] used by coxsackie B group of enteroviruses.

However, in the comparison of the different donor groups we acknowledge several limitations: (1) the limited number of donors positive for enterovirus RNA; (2) the varied time interval between AAb conversions or diagnosis and death; (3) sampling limitations, since the disease process occurs without uniformity across the pancreas; and (4) the extremely low amounts of virus may have caused stochastic variation in the amplification of viral RNA using RT-PCR. Additional variation could be caused by pancreatic enzymes and other compounds that degrade RNA, particularly when the viral RNA is present in unencapsidated forms where it is not protected by capsid proteins. This may make it difficult to detect a persisting enterovirus infection in pancreas [45], since such viruses replicate slowly and generate much-reduced amounts of complete virions compared with the acute infection [46]. Given these limitations, especially the low amount virus and positive samples, and as there is no clear sex bias reported in type 1 diabetes, sex analysis was not performed. When evaluating the RNA quality in a subset of pancreas, spleen and duodenal samples, variation in RNA degradation levels was evident. However, poor RQN values did not seem to affect the detection of enterovirus sequences by RT-PCR. This is not surprising, since the employed RT-PCR methods amplify very short fragments of viral RNA (in the range of 90–200 bp). The impact of RNA degradation is expected to be greater for RNA-Seq methods.

This study shows, for the first time, that enteroviruses can be found in PLNs as well. In fact, this fits well with their draining function as the lymph flow from the infected pancreas can carry virus to the nodes. Enterovirus RNA was also found in the spleen, another important lymphoid organ. Previous studies have shown elevated enterovirus titres in spleen during acute infection in humans and in animals [47–49], and in mouse models the spleen is enterovirus positive during a later phase of infection [44, 46, 50]. Detection of enterovirus RNA in spleen suggests infection of lymphoid cells. This is also supported by our own finding showing that enterovirus RNA can be detected using in situ RNA probes in immune cells infiltrating islets in the pancreas of individuals with type 1 diabetes [51]. We conducted co-culture studies in which spleen-derived enteroviruses caused no cytopathic effects in cell culture; this behaviour was previously seen with persistent enterovirus strains and linked to replication-defective viruses, including studies showing that CVB3 can lead to persistent and replication-defective infection in the mouse pancreas, accompanied by deletion in the 5′-non-coding region of the viral genome that regulates viral replication [14, 52].

Progression of the infection in our co-culture studies was slow and <5% cells stained positive for enterovirus VP1 (Fig. 3c–e), further supporting the presence of strains that produce persisting infection and/or replication-defective viruses in the spleen. The detection of virus in the lymphoid tissues of donors with type 1 diabetes raises important questions: which cell types are enterovirus positive in spleen (presumably of lymphoid nature); and how the virus may alter the function of infected cells. Previous studies have shown that enterovirus can infect human and murine leukocytes [53–55]. In our recent study of individuals with type 1 diabetes and control individuals, enterovirus RNA was found significantly more often in peripheral blood mononuclear cell (PBMC) subsets than in plasma and virus detection correlated with islet autoimmunity and the *IFIH1* genotype [56].

Enterovirus RNA was not detected in duodenal samples, in contrast to the findings of a previous study wherein both enterovirus RNA and VP1 protein were detected in duodenal biopsies taken from living individuals who had type 1 diabetes or were near diabetes diagnosis [16]. In addition, enterovirus VP1 was detected in formalin-fixed paraffin-embedded duodenal samples collected from these same nPOD donors as described in the accompanying publication by Rodriguez-Calvo et al [22]. On the other hand, a previous study failed to find enterovirus RNA in duodenal biopsies taken from individuals with type 1 diabetes but detected enterovirus protein at low frequency [18]. The reason for these discrepancies is unknown. Methodological differences, variation in the selection of cases, sample preparation and limited sampling may be involved. For example, previous studies have been based on biopsy samples collected from living individuals while the present study examined tissues from cadaver organ donors. Unlike pancreas tissue from organ donors, duodenal specimens are not perfused with tissue preservation buffer after harvesting, to protect from cold ischaemia. Thus, the unfixed duodenal tissue may not have been optimally preserved during transport, leading to partial RNA degradation.

A limitation of our study is that the RT-PCR approach did not allow the identification of the infected cell types in pancreas or other organs. Thus, the cell types harbouring enterovirus RNA remain unknown. A previous study that used in situ hybridisation in tissue sections showed enterovirus RNA in pancreatic islets of some donors with type 1 diabetes [4]. Another study showed enterovirus RNA by RT-PCR in cultured islets that had been isolated from living individuals with type 1 diabetes [6]. A recent study of nPOD donors found enterovirus RNA in both insulin-positive cells and insulin-negative cells in some donors with type 1 diabetes, including immune cells in the pancreas using fluorescent in situ hybridisation [51]. It is well known, and further corroborated by the accompanying nPOD-Virus group paper [22], that enterovirus VP1 is found primarily in insulin-positive beta cells and immune cells in the spleen. These cells are quite rare and this may explain the technical difficulties in detecting enterovirus RNA in pancreas [1]. An additional limitation is that the exact virus genotypes could not be determined since RT-PCR amplified a highly conserved genome region. However, we propose that even partial identification is valuable as it further links enterovirus infections to the pancreas and type 1 diabetes. It should also be noted that some control individuals who did not have type 1 diabetes and who were negative for islet AAbs were positive for enterovirus RNA in the pancreas, spleen or lymph nodes. This is not surprising given the high prevalence of enterovirus infections in the general population [57]. Additional data from our group suggest that host responses may be critical, among other factors (genetics, virus variants), in modulating the outcome of low-grade enterovirus infections in the pancreas.

Together with other studies of the nPOD-Virus Group, the present findings demonstrate that enterovirus RNA is present in organ donors with islet autoimmunity and ICIs, whether at the preclinical stage or after diagnosis, with an increased frequency compared with donors without diabetes. Moreover, donors with a single AAb had the highest prevalence of detection, consistent with enterovirus infections occurring early in the natural history of the disease. Despite limitations, the data support an association of enterovirus RNA with islet autoimmunity and suggest a low-grade enteroviral infection of pancreatic and lymphoid tissues.

## Supplementary Information

Below is the link to the electronic supplementary material.

ESM (PDF 166 KB)

## Data Availability

Data generated and analysed during this study are available through the corresponding author upon request.

## Abbreviations

AAb: Autoantibody
BCM: Baylor College of Medicine
CAR: Coxsackie and adenovirus receptor
CVB1: Coxsackie B virus 1
ds: Double-stranded
ICI: Insulin-containing islet
IDI: Insulin-deficient islet
ND: Non-diabetic
nPOD: Network for Pancreatic Organ Donors with Diabetes
PLN: Pancreatic lymph node
RQN: RNA quality number
T1D-ICI: Type 1 diabetes with insulin-containing islets (group)
T1D-IDI: Type 1 diabetes with insulin-deficient islets (group)
UCL: University College London
VP1: Viral capsid protein 1
WGS: Whole-genome shotgun
