# Supplementary material for: Detection of enterovirus RNA in pancreas and lymphoid tissues of organ donors with type 1 diabetes
Source: Diabetologia. 2025 Mar 17;68(6):1211–25. doi: 10.1007/s00125-025-06359-w (PMC12069483; doi:10.1007/s00125-025-06359-w)
Supplement: Supplementary file 1 — ESM (PDF 166 KB) [file 125_2025_6359_MOESM1_ESM.pdf]

Electronic supplementary material

Detection of enterovirus RNA in pancreas and lymphoid tissues of organ donors with type 1 diabetes

Laiho JE et al

ESM Methods. Description of the positive control experiment

Description of the preparation of the positive control samples for UCL RNA-Seq studies

An enterovirus (EV)-negative pancreas sample of a non-diabetic organ donor from the PanFin study [Tauriainen et al 2010] was homogenized using a Silent Crusher S homogenizer (Heidolph, Schwabach, Germany). The pancreas extract was divided into aliquots and spiked with infectious virus preparations of coxsackievirus B1 (CBV1). The virus was propagated in green monkey kidney (GMK) cells, and cell culture supernatant was used as virus source. Pancreas samples were spiked with virus dilutions (10^{-3}, 10^{-6}, 10^{-7}, 10^{-8}, 10^{-9}) and immediately frozen at −80 °C. Schematic presentation of the CVB1 spiked in pancreas dilution series used as positive control experiment for RNA-Seq analyses in UCL is presented below:

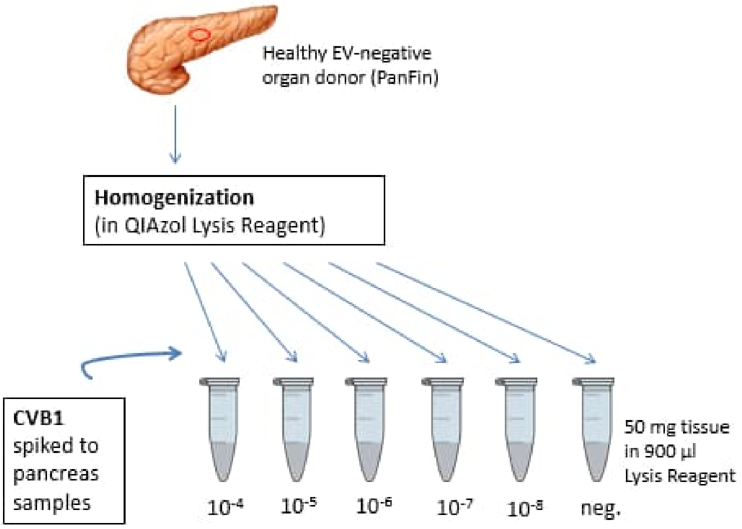

Ref. Tauriainen, S.; Salmela, K.; Rantala, I.; Knip, M.; Hyöty, H. Collecting high-quality pancreatic tissue for experimental study from organ donors with signs of beta-cell autoimmunity. *Diabetes Metab. Res. Rev.* **2010**, *26*, 585–592.

ESM Table 1: Donor demographics including RRiD, age, sex, BMI, c-peptide, duration of disease, AAb status, Ethnicity and the tissue type studied (ND= non-diabetic; AAb+= single autoantibody-positive; AAb++= multiple autoantibody-positive; T1D-ICI= type 1 diabetes with insulin-containing islets; T1D-IDI= type 1 diabetes with insulin-deficient islets; nd= not detected; NA= not applicable; Yrs= years; Y= yes)

Study Number	RRiD	Donor Type	Age (yrs)	Sex	BMI (kg/m2)	C-peptide (nmol/l)	Disease Duration (yrs)	Autoantibodies (Aabs)	Ethnicity	EV-PCR Pancreas	EV-PCR Spleen	EV-PCR Duodenum	EV-PCR PLN	RNA-Seq Pancreas	EV-Propagation Spleen
6005	SAMN15879062	ND	5	Female	15.7		NA	NA	White		Y				
6009	SAMN15879066	ND	45	Male	30.6	3.75	NA	NA	White	Y					
6010	SAMN15879067	ND	47	Female	19.7		NA	NA	White	Y	Y				
6012	SAMN15879069	ND	68	Female	23.7	0.98	NA	NA	White	Y				Y	
6013	SAMN15879070	ND	65	Male	24.2	0.93	NA	NA	White	Y					
6016	SAMN15879073	ND	64	Female	31.2		NA	NA	White	Y					
6017	SAMN15879074	ND	59	Female	24.8	3.27	NA	NA	White	Y				Y	
6019	SAMN15879076	ND	42	Male	31	0.16	NA	NA	White	Y				Y	
6020	SAMN15879077	ND	60	Male	29.8	0.93	NA	NA	White	Y					
6022	SAMN15879079	ND	75	Male	30.6	1.65	NA	NA	White	Y					
6024	SAMN15879081	ND	21	Male	27.8	1.17	NA	NA	White	Y	Y			Y	
6029	SAMN15879086	ND	24	Female	22.6		NA	NA	Hispanic	Y	Y				
6030	SAMN15879087	ND	30.1	Male	27.1	0.84	NA	NA	White	Y	Y			Y	
6034	SAMN15879091	ND	32	Female	25.2	1.04	NA	NA	White	Y	Y			Y	
6047	SAMN15879104	ND	7.8	Male	23.9	0.22	NA	NA	White	Y				Y	Y
6073	SAMN15879130	ND	19.2	Male	36	0.23	NA	NA	White	Y				Y	
6075	SAMN15879132	ND	16	Male	14.9	0.97	NA	NA	African Am	Y				Y	
6095	SAMN15879152	ND	40	Male	35.5		NA	NA	Hispanic	Y	Y	Y		Y	
6096	SAMN15879153	ND	16	Female	18.8	0.98	NA	NA	African Am	Y	Y	Y		Y	
6097	SAMN15879154	ND	43.1	Female	36.4	5.55	NA	NA	White	Y	Y	Y			
6098	SAMN15879155	ND	17.8	Male	22.8	0.47	NA	NA	White	Y				Y	
6099	SAMN15879156	ND	14.2	Male	30	1.78	NA	NA	White	Y				Y	
6102	SAMN15879159	ND	45.1	Female	35.1	0.18	NA	NA	White	Y	Y	Y		Y	Y
6103	SAMN15879160	ND	1.5	Male	16.8	0.32	NA	NA	White	Y	Y	Y		Y	
6104	SAMN15879161	ND	41	Male	20.5	6.80	NA	NA	White	Y	Y	Y		Y	Y

6106	SAMN15879163	ND	2.9	Male	17.4	2.44	NA	NA	White	Y	Y	Y			
6112	SAMN15879169	ND	6.3	Female	18.4	1.69	NA	NA	Hispanic	Y	Y	Y			
6117	SAMN15879174	ND	0.33	Male	18.4	1.08	NA	NA	White						Y
6126	SAMN15879183	ND	25.2	Male	25.1	0.29	NA	NA	Hispanic	Y	Y	Y		Y	
6130	SAMN15879187	ND	5.2	Male	18.5	1.59	NA	NA	White	Y	Y	Y			
6131	SAMN15879188	ND	24.2	Male	24.8	0.33	NA	NA	White	Y	Y	Y			
6137	SAMN15879194	ND	8.9	Female	24.2	4.02	NA	NA	Hispanic	Y	Y	Y		Y	Y
6140	SAMN15879197	ND	38	Male	21.7	3.68	NA	NA	White	Y	Y	Y			Y
6160	SAMN15879216	ND	22.1	Male	23.9	0.13	NA	NA	White	Y	Y	Y		Y	Y
6162	SAMN15879218	ND	22.7	Male	28.9	2.52	NA	NA	African Am	Y	Y	Y			
6165	SAMN15879221	ND	45.8	Female	25	1.47	NA	NA	White	Y	Y	Y		Y	
6168	SAMN15879224	ND	51	Male	25.2		NA	NA	Hispanic	Y	Y	Y			
6172	SAMN15879228	ND	19.2	Female	32.4	2.66	NA	NA	White	Y	Y	Y			
6174	SAMN15879230	ND	20.9	Male	19.5	0.99	NA	NA	White	Y	Y	Y			
6178	SAMN15879234	ND	24.5	Female	27.5	1.51	NA	NA	White	Y					Y
6179	SAMN15879235	ND	20	Female	20.7	0.91	NA	NA	White	Y	Y	Y			
6182	SAMN15879238	ND	2.7	Male	26	0.75	NA	NA	White	Y	Y	Y		Y	Y
6190	SAMN15879246	ND	0.83	Male	14.2	1.82	NA	NA	Hispanic						Y
6227	SAMN15879283	ND	17	Female	26.4	0.91	NA	NA	White						Y
6238	SAMN15879294	ND	20	Male	21.7	0.39	NA	NA	African Am	Y	Y				
6254	SAMN15879310	ND	38	Male	30.5	2.13	NA	NA	White	Y	Y				Y
6278	SAMN15879332	ND	12	Female	21.3	1.50	NA	NA	African Am	Y	Y				
6282	SAMN15879336	ND	14	Male	41.9	2.26	NA	NA	White						Y
6289	SAMN15879343	ND	19	Male	38.3	2.67	NA	NA	African Am						Y
6295	SAMN15879349	ND	47	Female	30.4	3.61	NA	NA	African Am						Y
6318	SAMN15879372	ND	10	Female	17.6	1.29	NA	NA	White						Y
6333	SAMN15879387	ND	27.1	Female	24.9	3.10	NA	NA	White						Y
6338	SAMN15879392	ND	16.9	Male	22.8	1.66	NA	NA	African Am						Y
6339	SAMN15879393	ND	23.2	Male	25	3.50	NA	NA	White	Y	Y				
6340	SAMN15879394	ND	9.7	Male	20.3	1.28	NA	NA	White						Y
6350	SAMN15879404	ND	3.3	Female	12.7	0.73	NA	NA	Hispanic						Y
6353	SAMN15879406	ND	13	Male	28.3	0.58	NA	NA	African Am						Y

6356	SAMN15879409	ND	1.58	Female	17.1	0.55	NA	NA	White						Y
6357	SAMN15879410	ND	5	Male	15.3	2.92	NA	NA	White						Y
6364	SAMN15879417	ND	4.6	Male	18	1.09	NA	NA	Hispanic						Y
6366	SAMN15879419	ND	21	Female	20.5	0.14	NA	NA	Hispanic						Y
6368	SAMN15879421	ND	38.3	Male	20.7	1.01	NA	NA	White						Y
6369	SAMN15879422	ND	44	Male	18.8	2.13	NA	NA	White						Y
6375	SAMN15879428	ND	28.7	Male	31.8	5.73	NA	NA	White	Y	Y				Y
6381	SAMN15879434	ND	6.6	Male	22.6	1.85	NA	NA	African Am						Y
6384	SAMN15879437	ND	17	Male	18.2	0.23	NA	NA	White	Y	Y				
6385	SAMN15879438	ND	10.9	Male	16.2	0.51	NA	NA	White						Y
6386	SAMN15879439	ND	14	Male	23.9	0.37	NA	NA	White						Y
6401	SAMN15879454	ND	25.07	Female	31.3	4.24	NA	NA	Hispanic	Y	Y				
6406	SAMN15879459	ND	6.9	Male	16.8	1.35	NA	NA	White	Y	Y				Y
6407	SAMN15879460	ND	4.6	Female	16	1.77	NA	NA	White						Y
6412	SAMN15879465	ND	17.6	Female	24	15.09	NA	NA	White						Y
6413	SAMN15879466	ND	10.1	Female	19	1.74	NA	NA	White	Y	Y				Y
6420	SAMN15879473	ND	11.5	Male	15.4	0.42	NA	NA	White						Y
6027	SAMN15879084	AAb$^+$	18.8	Male	19.9		NA	ZnT8A+	White	Y				Y	
6044	SAMN15879101	AAb$^+$	41.4	Male	27.4	4.49	NA	GADA+	Hispanic	Y	Y			Y	
6090	SAMN15879147	AAb$^+$	2.2	Male	18.8	1.77	NA	GADA+	Hispanic	Y	Y	Y		Y	
6101	SAMN15879158	AAb$^+$	64.8	Male	34.3	8.67	NA	GADA+	White	Y	Y	Y		Y	
6123	SAMN15879180	AAb$^+$	23.2	Female	17.6	0.67	NA	GADA+	White	Y	Y	Y		Y	Y
6147	SAMN15879203	AAb$^+$	23.8	Female	32.9	1.06	NA	GADA+	White	Y				Y	
6151	SAMN15879207	AAb$^+$	30	Male	24.2	1.82	NA	GADA+	White	Y	Y	Y		Y	Y
6154	SAMN15879210	AAb$^+$	48.5	Female	24.5	0.02	NA	GADA+	White	Y	Y	Y		Y	
6156	SAMN15879212	AAb$^+$	40	Male	19.8	4.42	NA	GADA+	White	Y	Y	Y		Y	Y
6171	SAMN15879227	AAb$^+$	4.4	Female	14.8	2.96	NA	GADA+	White	Y	Y			Y	Y
6181	SAMN15879237	AAb$^+$	31.9	Male	21.9	0.02	NA	GADA+	White	Y	Y	Y		Y	Y
6184	SAMN15879240	AAb$^+$	47.6	Female	27	1.13	NA	GADA+	Hispanic	Y	Y	Y		Y	
6314	SAMN15879368	AAb$^+$	21	Male	23.8	0.49	NA	GADA+	White	Y					
6400	SAMN15879453	AAb$^+$	25.15	Male	22.2	1.38	NA	GADA+	Hispanic	Y	Y				

6421	SAMN15879474	AAb^{+}	6.73	Male	17.9	0.61	NA	GADA+	Hispanic	Y	Y				Y
6080	SAMN15879137	AAb^{++}	69.2	Female	21.3	0.61	NA	mIAA+ GADA+	White	Y	Y	Y	Y	Y	Y
6158	SAMN15879214	AAb^{++}	40.3	Male	29.7	0.17	NA	mIAA+ GADA+	White	Y	Y	Y	Y	Y	Y
6167	SAMN15879223	AAb^{++}	37	Male	26.3	1.80	NA	IA2A+ ZnT8A+	White	Y	Y	Y	Y	Y	
6197	SAMN15879253	AAb^{++}	22	Male	28.2	5.79	NA	GADA+ IA2A+	African Am	Y	Y	Y		Y	
6267	SAMN15879321	AAb^{++}	23	Female	23.5	5.49	NA	GADA+ IA2A+	White	Y	Y	Y			
6424	SAMN15879477	AAb^{++}	17.65	Male	51.4	2.31	NA	mIAA+ GADA+	Hispanic	Y	Y				
6429	SAMN15879482	AAb^{++}	22.1	Male	19.6	0.74	NA	mIAA+ GADA+	African Am	Y	Y				
6038	SAMN15879095	T1D-ICI	37.2	Female	30.9	0.07	20	Negative	White		Y				
6046	SAMN15879103	T1D-ICI	18.8	Female	25.2	nd	8	GADA+ ZnT8A+	White	Y	Y			Y	Y
6049	SAMN15879106	T1D-ICI	15	Female	20.8	nd	10	GADA+ mIAA+	African Am	Y					
6051	SAMN15879108	T1D-ICI	20.3	Male	21.5	nd	13	mIAA+	White	Y				Y	
6052	SAMN15879109	T1D-ICI	12	Male	20.3	0.06	1	GADA+ mIAA+	African Am	Y	Y			Y	Y
6070	SAMN15879127	T1D-ICI	22.6	Female	21.6	nd	7	GADA+ mIAA+	White	Y	Y	Y		Y	
6084	SAMN15879141	T1D-ICI	14.2	Male	26.3	nd	4	mIAA+	White	Y	Y			Y	Y
6088	SAMN15879145	T1D-ICI	31.2	Male	27	nd	5	GADA+ IA2A+ mIAA+ ZnT8A+	White	Y	Y	Y		Y	
6113	SAMN15879170	T1D-ICI	13.1	Female	24.75	nd	1.58	mIAA+	White	Y	Y	Y		Y	Y
6180	SAMN15879236	T1D-ICI	27.1	Male	25.9	nd	11	GADA+ IA2A+ mIAA+ ZnT8A+	White	Y				Y	Y
6195	SAMN15879251	T1D-ICI	19.3	Male	23.7	nd	5	GADA+ IA2A+ mIAA+ ZnT8A+	White	Y	Y	Y		Y	Y
6196	SAMN15879252	T1D-ICI	26.5	Female	26.6	0.16	15	GADA+ mIAA+	African Am	Y	Y	Y			
6198	SAMN15879254	T1D-ICI	22	Female	23.1	nd	3	GADA+ IA2A+ mIAA+ ZnT8A+	Hispanic	Y	Y			Y	
6209	SAMN15879265	T1D-ICI	5	Female	15.9	0.03	0.25	IA2A+ mIAA+ ZnT8A+	White	Y	Y	Y	Y	Y	
6211	SAMN15879267	T1D-ICI	24	Female	24.4	nd	4	GADA+ IA2A+ mIAA+ ZnT8A+	African Am	Y	Y	Y		Y	Y
6212	SAMN15879268	T1D-ICI	20	Male	29.1	nd	5	mIAA+	White	Y				Y	Y
6228	SAMN15879284	T1D-ICI	13	Male	17.4	0.03	0	GADA+ IA2A+ ZnT8A+	White	Y			Y	Y	
6243	SAMN15879299	T1D-ICI	13	Male	21.3	0.14	5	mIAA+	White					Y	Y
6245	SAMN15879301	T1D-ICI	22	Male	23.2	nd	7	GADA+ IA2A+	White	Y				Y	
6247	SAMN15879303	T1D-ICI	24	Male	24.3	0.16	0.6	mIAA+	White	Y	Y	Y	Y		
6264	SAMN15879318	T1D-ICI	12	Female	22	nd	9	Negative	White	Y					
6265	SAMN15879319	T1D-ICI	11	Male	12.9	0.02	8	GADA+ mIAA+	White	Y					Y

6268	SAMN15879322	T1D-ICI	12	Female	26.6	0.02	3	mIAA+	White						Y
6302	SAMN15879356	T1D-ICI	38.5	Male	20.5	0.06	32.5	Negative	African Am	Y					
6306	SAMN15879360	T1D-ICI	19	Male	24.5	nd	5	mIAA+	White	Y					Y
6307	SAMN15879361	T1D-ICI	45	Female	19.5	nd	10	GADA+ mIAA+	White	Y					
6325	SAMN15879379	T1D-ICI	20	Female	31.2	0.05	6	GADA+ IA2A+ mIAA+	African Am	Y					Y
6328	SAMN15879382	T1D-ICI	39	Male	24	nd	20	GADA+ mIAA+	Hispanic	Y					
6337	SAMN15879391	T1D-ICI	20.6	Female	17.9	nd	5	mIAA+	White	Y					
6342	SAMN15879396	T1D-ICI	14	Female	24.3	0.09	2	IA2A+ mIAA+	White	Y	Y	Y			Y
6362	SAMN15879415	T1D-ICI	24.9	Male	28.5	0.13	0	GADA+	White	Y	Y				Y
6367	SAMN15879420	T1D-ICI	24	Male	25.7	0.13	2	Negative	White	Y	Y				Y
6371	SAMN15879424	T1D-ICI	12.5	Female	16.6	0.04	2	GADA+ IA2A+ mIAA+ ZnT8A+	White	Y	Y				
6380	SAMN15879433	T1D-ICI	11.6	Female	14.6	0.07	0	Negative	African Am	Y	Y				
6405	SAMN15879458	T1D-ICI	29.1	Female	42.5	0.61	0.6	GADA+ IA2A+ ZnT8A+	Hispanic	Y	Y				
6026	SAMN15879083	T1D-IDI	22.4	Male	24.1	nd	9	mIAA+	White	Y				Y	
6031	SAMN15879088	T1D-IDI	39	Male	24.5	nd	35	mIAA+	White	Y					
6035	SAMN15879092	T1D-IDI	32.1	Male	27.1	nd	28	mIAA+	White	Y	Y				
6039	SAMN15879096	T1D-IDI	28.7	Female	23.4	nd	12	GADA+ IA2A+ mIAA+ ZnT8A+	White	Y				Y	
6041	SAMN15879098	T1D-IDI	26.3	Male	28.4	nd	23	Negative	White	Y					
6045	SAMN15879102	T1D-IDI	26.4	Male	23.1	nd	8	mIAA+ ZnT8A+	White	Y				Y	
6063	SAMN15879120	T1D-IDI	4.4	Male	23.8	nd	3	mIAA+	White	Y	Y	Y		Y	Y
6066	SAMN15879123	T1D-IDI	78	Male	30.9	nd	74	IA2A+ mIAA+	White	Y					
6067	SAMN15879124	T1D-IDI	32.6	Female	26.8	nd	8	Negative	Hispanic	Y					
6076	SAMN15879133	T1D-IDI	25.8	Male	18.8	nd	15	GADA+ mIAA+	White	Y				Y	
6077	SAMN15879134	T1D-IDI	32.9	Female	22	nd	19	mIAA+	White	Y				Y	
6079	SAMN15879136	T1D-IDI	11.1	Female	18.6	nd	8	Negative	White	Y					Y
6083	SAMN15879140	T1D-IDI	15.2	Female	18.4	nd	11	mIAA+	White	Y	Y	Y			
6087	SAMN15879144	T1D-IDI	17.5	Male	21.9	nd	4	mIAA+ ZnT8A+	White	Y	Y	Y			
6089	SAMN15879146	T1D-IDI	14.3	Male	26	nd	8	mIAA+	White	Y	Y	Y			
6119	SAMN15879176	T1D-IDI	7.8	Male	19.4	nd	14	GADA+ mIAA+	White	Y				Y	Y
6128	SAMN15879185	T1D-IDI	33.8	Female	22.2	nd	31.5	mIAA+	White	Y	Y	Y		Y	Y
6135	SAMN15879192	T1D-IDI	43.5	Male	28.7	nd	21	GADA+ mIAA+	White	Y	Y	Y			

6138	SAMN15879195	T1D-IDI	49.2	Female	33.7	nd	41	mIAA+	White	Y	Y	Y			
6141	SAMN15879198	T1D-IDI	36.7	Male	26	nd	28	GADA+ IA2A+ mIAA+ ZnT8A+	White	Y	Y	Y		Y	
6143	SAMN15879200	T1D-IDI	32.6	Female	26.1	nd	7	IA2A+ mIAA+	White	Y	Y	Y		Y	Y
6145	SAMN15879202	T1D-IDI	18	Male	23.1	0.02	11	GADA+ mIAA+ ZnT8A+	White	Y	Y	Y			
6148	SAMN15879204	T1D-IDI	17.1	Male	23.9	nd	7	GADA+ mIAA+	White	Y	Y	Y			Y
6152	SAMN15879208	T1D-IDI	29.6	Female	30.1	nd	12	ZnT8A+	White	Y	Y	Y			
6155	SAMN15879211	T1D-IDI	50	Female	26	nd	43	mIAA+	White	Y	Y	Y			
6159	SAMN15879215	T1D-IDI	50.8	Female	35.5	nd	44	mIAA+	White	Y	Y	Y			
6161	SAMN15879217	T1D-IDI	19.2	Female	36.1	nd	7	IA2A+ mIAA+	White	Y	Y	Y			Y
6163	SAMN15879219	T1D-IDI	32.5	Male	25.5	nd	30	IA2A+ mIAA+	White	Y	Y	Y			
6169	SAMN15879225	T1D-IDI	27.6	Female	25	nd	15	GADA+ mIAA+	Hispanic	Y	Y	Y			
6173	SAMN15879229	T1D-IDI	44.1	Male	23.9	nd	15	Negative	White	Y	Y	Y			
6205	SAMN15879261	T1D-IDI	40.9	Female	22.6	0.05	33	mIAA+	White	Y	Y	Y			
6207	SAMN15879263	T1D-IDI	16.7	Female	24.4	nd	10	IA2A+ mIAA+ ZnT8A+	African Am	Y	Y	Y			
6208	SAMN15879264	T1D-IDI	32.6	Female	23.4	nd	16	Negative	White	Y	Y	Y			
6224	SAMN15879280	T1D-IDI	21	Female	22.8	nd	1.5	Negative	White				Y		Y
6324	SAMN15879378	T1D-IDI	29	Male	26.2	nd	2	GADA+ mIAA+	Hispanic	Y	Y	Y	Y		
6418	SAMN15879471	T1D-IDI	24.9	Male	26.4	nd	11	GADA+, IA-2A+, mIAA+*, ZnT8+	White						Y

ESM Table 2: Detection of CVB1 specific sequences by metagenomic sequencing in homogenised pancreas samples spiked with different dilutions with CVB1 (five spiked-in samples with CVB1 and one negative un-spiked control sample).

	Raw	QC	QC	CVB1 aligned	CVB1
spike-in dilution of CVB1	# pairs	# pairs	%	# reads	%
0 (negative control)	4728119	4349094	0.92	2reads->1 pair	
$1x10^{-8}$	4223286	3971535	0.94	32	4.0×10^{-6}
$1x10^{-7}$	3710861	3351818	0.90	283	4.2×10^{-5}
$1x10^{-6}$	3546996	3229128	0.91	2595	4.0×10^{-4}
$1x10^{-5}$	4392146	4015026	0.91	12237	1.5×10^{-3}
$1x10^{-4}$	2448108	2149775	0.88	132770	3.1×10^{-2}

ESM Table 3. UCL Step I RNA-seq analyses from nPOD samples: Taxonomic classification of the "microbial" reads with metaMix resulted in a similar profile for all samples, showing no difference between case and control donors. The majority of reads was assigned to Enterobacteria phage phiX174, the positive control for Illumina sequencing. The rest of the reads were divided between various environmental bacteria and the “unknown” bin. Table represents, as an example, a general profile and relative abundances in 6070 (T1D-ICI), 6098 (ND), 6141 (T1D-IDI).

Organisms	mean abundance % (sd)
Enterobacteria phage phiX174	62 (0.03)
Environmental bacteria	25 (0.02)
Unknown	13 (0.01)

T1D-ICI – type 1 diabetes with insulin containing islets ; T1D-IDI – type 1 diabetes with isulin deficient islets ; ND – non-diabetic

ESM Table 4: Example of metaMix summary profile for one case, sequenced using the sequence capture approach to enrich enterovirus specific sequences (Step III).

taxon id	scientific name	assigned reads	posterior prob
unknown	unknown	188927	1
9606	*Homo sapiens*	2745	1
374840	*Enterobacteria phage phiX174 sensu lato*	698	0.92

ESM Table 5. Group comparisons of enterovirus positivity in the pancreas by RT-PCR (relates to **Fig.2 A**)

**Donor group comparisons**	**P value (Fisher exact test 2-sided)**	**P value corrected (10 comparisons)**
**ND vs Aab^{+}**	**0.0004**	**0.004**
ND vs Aab^{++}	1.000	N.S.
ND vs T1D-ICI	0.4713	N.S.
ND vs T1D-IDI	0.1405	N.S.
Aab+ vs Aab^{++}	0.0225	N.S.
Aab+ vs T1D-ICI	**0.0131**	N.S.
**Aab+ vs T1D-IDI**	**0.000**	**0.000**
Aab++ vs T1D-ICI	0.5628	N.S.
Aab++ vs T1D-IDI	1.000	N.S.
**T1D-ICI vs T1D-IDI**	**0.0225**	N.S.

ESM Table 6. Pancreas enterovirus positivity with regards to active islet autoimmunity (IA) (relates to **Fig.2 B**). Significant p-values are bolded.

**Donor group comparisons**	**P value (Fisher exact test 2-sided)**	**P value corrected (6 comparisons)**
ND vs IA and ICIs	**0.0078**	**0.0468**
ND vs Autoimmunity	**0.0639**	N.S.
Autoimmunity vs Autoimmunity and ICIs	0.367	N.S.
Autoimmunity and ICIs vs No autoimmunity	**0.001**	**0.006**
ND vs No autoimmunity	0.2907	N.S.
Autoimmunity vs No autoimmunity	**0.004**	**0.04**

ESM Table 7. Enterovirus detection in the spleen **by RT-PCR**. Corrected p-values for multiple comparisons (N=10) are also shown (relates to **Fig. 3A**)

**Donor group comparisons**	**P value (Fisher exact 2-sided)**	**P value corrected (10 comparisons)**
ND vs Aab^{+}	0.5597	N.S.
ND vs Aab^{++}	1	N.S.
ND vs T1D-ICI	0.6823	N.S.
ND vs T1D-IDI	0.6392	N.S.
Aab^{+} vs Aab^{++}	0.3684	N.S.
Aab^{+} vs T1D-ICI	0.2645	N.S.
Aab^{+} vs T1D-IDI	1	N.S.
Aab^{++} vs T1D-ICI	1	N.S.
Aab^{++} vs T1D-IDI	0.4184	N.S.
T1D-ICI vs T1D-IDI	0.3129	N.S.

ESM Table 8. RNA quality numbers (RQN) for selected nPOD donors in different tissues vs. enterovirus positivity by RT-PCR.

		**Pancreas**		**Spleen**		**Duodenum**	
**Donor ID**	**Donor type**	**EV PCR**	**RQN**	**EV PCR**	**RQN**	**EV PCR**	**RQN**
6097	ND			POS	6.2	NEG	2.2
6044	Aab^{+}	POS	1				2
6046	T1D-ICI	POS	5.3	POS	1.1		
6087	T1D-IDI			POS	2.4		
6090	Aab^{+}	NEG	6.3				
6101	Aab^{+}	POS	2				
6102	ND					NEG	1.5
6106	ND					NEG	1
6112	ND			POS	1	NEG	1
6123	Aab^{+}	POS	2.8				
6154	Aab^{+}	POS	3				
6156	Aab^{+}	POS	5				
6158	Aab^{++}	POS	3.9				
6167	Aab^{++}	NEG	1.5				
6209	T1D-ICI	POS	5.7	POS	1.6	NEG	1.3
6247	T1D-ICI	NEG	7.8			NEG	4.4
6267	Aab^{++}	NEG	5.8			NEG	3.6
6324	T1D-IDI					NEG	7.3
6342	T1D-ICI					NEG	3

ESM Table 9. Enterovirus detection in the spleen by enterovirus propagation. Significant p-values are bolded. Corrected p-values for multiple comparisons (N=10) are also shown (Relates to **Fig. 2B**)

**Donor group comparisons**	**P value (Fisher exact 2-sided)**	**P value corrected***
ND vs Aab^{+}	1	N.S
ND vs Aab^{++}	1	N.S
ND vs T1D-ICI	**0.0069**	N.S
ND vs T1D-IDI	**0.001**	**0.01**
Aab^{+} vs Aab^{++}	1	N.S
Aab^{+} vs T1D-ICI	0.1619	N.S
Aab^{+} vs T1D-IDI	0.0406	N.S
Aab^{++}vs T1D-ICI	0.4706	N.S
Aab^{++} vs T1D-IDI	0.1091	N.S
T1D-ICI vs T1D-IDI	0.4013	N.S

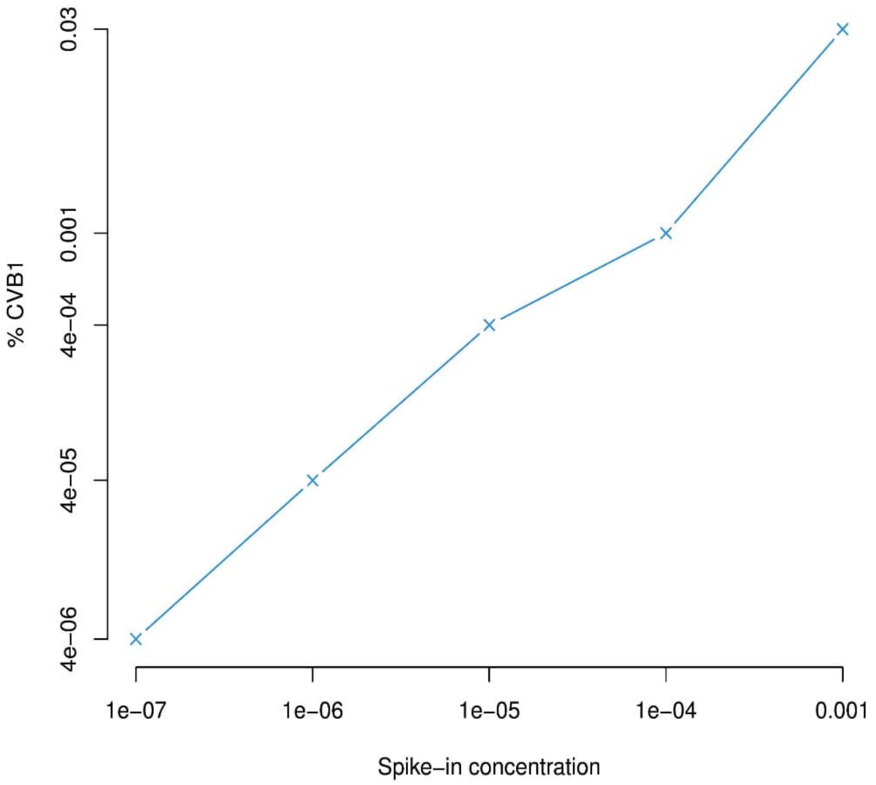

ESM. Fig. 1. Linear relationship between CVB1 spike-in concentrations and % on the target viral reads. Both axes are in log10 scale.

nPOD-V Funding 2012
Funding Reference: Breakthrough T1D (formally JDRF) JDRF-25-2012-516
Participants:
Pugliese, Alberto, M.D. Arthur Riggs Diabetes & Metabolism Research Institute, City of Hope, Duarte, CA, USA Principal Investigator
Atkinson, Mark, Ph.D. University of Florida Sr. Advisor
Campbell-Thompson, Martha University of Florida Investigator
Chapman, Nora, Ph.D. University of Nebraska Investigator
Coppieters, Ken University of Ghent Investigator
Dotta, Francesco, M.D. University of Siena Investigator
Eisenbarth, George, M.D., Ph.D. Barbara Davis Center for Childhood Diabetes Sr. Advisor
Ferreira, Ricardo, Ph.D JDRF/WT Diabetes & Inflammation Laboratory Investigator (Post-Doc)
Frisk, Gun, Ph.D. University of Uppsala Investigator
Gianani, Roberto, M.D. Barbara Davis Center for Childhood Diabetes Investigator
Gerling, Ivan, Ph.D University of Tennessee Investigator
Homann, Dirk, M.D. University of Colorado at Denver Investigator
Hyoty, Heikki, Ph.D. Tampere University, Finland Investigator
Lloyd, Richard, Ph.D. Baylor College of Medicine Investigator
Kaddis, John, Ph.D. City of Hope National Medical Center Investigator
Kent, Sally, Ph.D. University of Massachusetts Investigator
Morgan, Noel, Ph.D. University of Exeter, UK Investigator
Nadler, Jerry, M.D., Ph.D. East Virginia Medical School Investigator
Morris Fear, Margareta, Ph.D. East Virginia Medical School Investigator
Nyalwhide, Julius, Ph.D. East Virginia Medical School Investigator
Oikarinen, Maarit Tampere University, Finland Research Scientist
Plagnol, Vincent, Ph.D. University College of London Investigator
Petrosino, Joseph, Ph.D. Baylor College of Medicine Investigator
Richardson, Sarah, Ph.D. University of Exeter, UK Investigator (Post-Doc)
Sarkar, Suparna Barbara Davis Center for Childhood Diabetes Investigator
Schneider, Darius La Jolla Inst. Allergy & Immunology Investigator (Post-Doc)
Thackray, Larissa, Ph.D. Washington University Investigator
Toniolo, Antonio. M.D. University of Insubria *Investigator*
Virgin, Herbert Washington University Investigator
Von Herrath, Matthias La Jolla Inst. Allergy & Immunology Investigator

nPOD-V Grant 2017
Funding Reference: Breakthrough T1D (formally JDRF) JDRF-3-SRA-2017-492-A-N
Participants:
Dr Alberto Pugliese, *Arthur Riggs Diabetes & Metabolism Research Institute, City of Hope, Duarte, CA, USA Principal Investigator*
Dr. Richard Lloyd, Baylor College of Medicine, USA.
Dr. Margaret Morris, Eastern Virginia Medical School, USA
Dr. Roberto Mallone, INSERM, Paris, France

Dr. Malin Flodström Tullberg, Karolinska Institutet, Sweden
Dr. Matthias von Herrath, La Jolla Institute for Allergy and Immunology, USA
Dr. Jerry Nadler, Eastern Virginia Medical School, USA
Dr. Julius Nyalwidhe, Eastern Virginia Medical School, USA
Dr. Maria Teresa Rodriguez Calvo, La Jolla Institute for Allergy and Immunology.
Dr. Sally Kent, University of Massachusetts Medical School, USA
Dr. Antonio Toniolo, University of Insubria, Italy
Dr. Kathrin Maedler, University of Bremen, Germany
Dr. Marc Horwitz, University of British Columbia, Canada
Dr. Noel Morgan, University of Exeter Medical School, UK
Dr. Sarah Richardson, University of Exeter Medical School, UK
Dr. Mark Atkinson, University of Florida, USA
Dr. Ivan Gerling, The University of Tennessee Health Science Center, USA
Dr. Heikki Hyoty, *Tampere University, Finland*
Dr. Isaac Snowhite, University of Miami School of Medicine, USA
Dr. Filippo Canducci, University of Insubria, Italy
Dr. Alessandro Salvatoni, University of Insubria*, Italy*

Additional members **(some participated in discussions, but their participation in the group is more informal).**
Soile Tuomela PhD (Karolinska Institutet, Sweden)
Ben Giepmans PhD (University of Groningen, Netherlands)
Varpu Marjomaki PhD (University of Jyväskylä, Finland)
